# Cancer Therapy Related Cardiotoxicity

**DOI:** 10.2174/157340311799960663

**Published:** 2011-11

**Authors:** Maysa M Abu-Khalaf, Feras Bader

In the last two decades, significant advances in cancer therapy development have lead to more effective chemotherapeutic and novel targeted therapies for cancer patients. Although this has resulted in a remarkable improvement in cure rates and cancer-related survival, it has also resulted in unwanted long term side effects in a growing population of survivors. In recent years, cardiac toxicity has been increasingly recognized as a potential complication of cancer therapy, and in some cases, risk factors have been identified. There are ongoing research efforts focused on the management, and more importantly the prevention of this serious complication. 

We are pleased to introduce this issue titled **“Cancer Therapy Related Cardiotoxicity”** with a focused update on the early recognition and management of this particularly concerning complication. Among the key clinical considerations, the authors describe the cardiotoxicity risk factors that should be recognized and considered when formulating a treatment plan for cancer patients. The authors discuss potential underlying pathophysiologic mechanisms which may result in better understanding of the cardiac manifestations resulting from various cancer treatments. In addition, they present modern diagnostic techniques as well as recommendations for a multidisciplinary approach between oncologists and cardiologist involved in the care of cancer patients. 

